# The regulatory role of IL-37 and IL-38 in CAR-T associated cytokine release syndrome in multiple myeloma

**DOI:** 10.3389/fimmu.2026.1882156

**Published:** 2026-07-14

**Authors:** Xiujuan Huang, Hailong Yan, Jiaojiao Bai, Shisan Bao, Yi Wang, Qiuying Gao

**Affiliations:** 1 Department of Hematology, Shaanxi Provincial People’s Hospital, Xi’an, China; 2 Department of Emergency Surgery, Shaanxi Provincial People’s Hospital, Xi’an, Shaanxi, China; 3 Center for Evidence-based Medicine, Gansu University of Chinese Medicine, Lanzhou, Gansu, China

**Keywords:** car-t, cytokine release syndrome, IL-37, IL-38, multiple myeloma

## Abstract

Multiple myeloma remains largely incurable despite advances in proteasome inhibitors and monoclonal antibodies. Chimeric antigen receptor (CAR)-T-cell therapy targeting B-cell maturation antigen (BCMA) has achieved deep responses in relapsed/refractory multiple myeloma; however, its clinical utility is constrained by cytokine release syndrome (CRS). CRS is a multicellular hyperinflammatory process driven by CAR-T-derived cytokines, monocyte/macrophage activation, and amplification of the IL-1β–IL-6 axis, leading to endothelial dysfunction and metabolic reprogramming. While IL-6 blockade is the standard of care, severe CRS often persists due to redundant upstream inflammatory signalling. This mini-review evaluates the emerging roles of IL-37 and IL-38, anti-inflammatory members of the IL-1 superfamily, as endogenous regulators of CAR-T-associated hyperinflammation. IL-37 functions primarily as a systemic mediator that suppresses NF-κB/MAPK signalling, inflammasome activity, and endothelial injury. In contrast, IL-38 acts as a tissue-resident regulator that restrains early innate immune priming and modulates macrophage-dendritic cell interactions within the bone marrow microenvironment. We propose a phase-dependent regulatory axis wherein IL-38 limits inflammatory initiation while IL-37 suppresses systemic amplification during peak CRS. These pathways represent promising immunoregulatory checkpoints with translational potential as biomarkers and therapeutic targets. Leveraging these cytokines through recombinant proteins or “armoured” CAR-T-cells equipped with inducible regulatory circuits may improve the safety and efficacy of cellular immunotherapies in multiple myeloma.

## Clinical burden of multiple myeloma

Multiple myeloma, the second most common haematological malignancy, is characterised by clonal proliferation of malignant plasma cells within the bone marrow and the development of a profoundly immunosuppressive tumour microenvironment (1). Clinically, multiple myeloma drives progressive end-organ damage manifested by osteolytic bone lesions, anaemia, renal impairment, and marked immune dysregulation (2). The disease typically follows a relapsing-remitting course driven by ongoing clonal evolution and dynamic interactions between malignant plasma cells and the bone marrow niche. Despite major therapeutic advances, including proteasome inhibitors (3), immunomodulatory drugs (4), and monoclonal antibodies, multiple myeloma remains largely incurable, with most patients eventually developing relapsed or refractory disease (5).

Although survival outcomes in multiple myeloma have improved substantially over the past two decades, most patients still require multiple sequential lines of therapy, with progressively shorter durations of response over time (6). This chronic disease trajectory imposes a considerable clinical burden through cumulative organ toxicity, impaired quality of life, and substantial psychological and caregiver stress (1).

From a health economics perspective, multiple myeloma is associated with persistently high treatment-related costs due to prolonged therapy, recurrent hospitalisation, and management of disease- and treatment-related complications (7). The introduction of chimeric antigen receptor T-cell (CAR-T) therapy has further intensified this burden because of its personalised manufacturing process, intensive inpatient monitoring requirements, and the management of severe immune-mediated toxicities (8, 9).

The current mini-review is intended as a hypothesis-generating, integrative review that highlights future research directions rather than proposing immediate clinical applications.

## Therapeutic landscape of multiple myeloma

The current management of multiple myeloma integrates several distinct therapeutic classes that target different aspects of disease biology and immune evasion. These include proteasome inhibitors such as bortezomib and carfilzomib (3), which disrupt protein homeostasis and induce endoplasmic reticulum stress in malignant plasma cells. Immunomodulatory drugs (IMiDs) such as lenalidomide and pomalidomide enhance anti-tumour immune activity through cereblon-mediated degradation of Ikaros and Aiolos while simultaneously exerting direct anti-myeloma effects (4). Treatment is further complemented by anti-CD38 monoclonal antibodies (10), which promote immune-mediated cytotoxicity *via* antibody-dependent cellular cytotoxicity and complement-dependent cytotoxicity, and autologous stem cell transplantation (ASCT), which remains a key consolidative strategy in eligible patients (11). Additionally, allogeneic adoptive transfer represents an alternative immunotherapeutic approach, although its broader clinical implementation has been constrained by factors including graft-versus-host disease, feasibility considerations, and the longstanding preference for autologous strategies (12).

More recently, the therapeutic landscape has expanded to include novel immunotherapies, particularly bispecific T-cell engagers and chimeric antigen receptor T-cell (CAR-T) therapies (1, 6). Among these emerging approaches, CAR-T therapy targeting B-cell maturation antigen (BCMA) represents a major breakthrough in relapsed or refractory multiple myeloma, achieving deep and durable responses even in heavily pretreated patients (6). Despite these remarkable clinical outcomes, response durability remains heterogeneous, and the broader application of CAR-T therapy is constrained by significant immune-related toxicities (9). In particular, cytokine release syndrome (CRS) is the most clinically relevant and frequently encountered adverse event, representing a major limitation to the safe and widespread implementation of CAR-T therapy in multiple myeloma (8).

## CAR-T-associated toxicities: beyond classical CRS

CAR-T-cell therapy has transformed the treatment landscape of multiple myeloma, particularly through targeting BCMA. However, its clinical efficacy is substantially limited by CRS, a potentially life-threatening systemic inflammatory toxicity (13, 14). CRS remains one of the most frequent and clinically significant complications of CAR-T-therapy, reflecting a highly dynamic and self-amplifying immune activation process that continues to challenge clinical management (15, 16).

Mechanistically, CRS is now understood as a multi-cellular inflammatory circuit rather than a T cell–restricted phenomenon. Following recognition of BCMA-expressing tumour cells, CAR-T-cells activate CD3ζ-dependent signalling, leading to robust secretion of IFNγ, TNF, and granulocyte–macrophage colony-stimulating factor (GM-CSF) (13). GM-CSF plays a pivotal role in linking adaptive and innate immunity by driving monocyte and macrophage activation, thereby initiating a secondary wave of cytokine release (17).

This cascade is further amplified within myeloid cells *via* JAK-STAT signalling, NF-κB activation, and NOD-like receptor family pyrin domain containing 3 (NLRP3) inflammasome assembly, culminating in the production of IL-1β and IL-6 (18). Importantly, IL-1β acts upstream to potentiate IL-6 expression, forming an autocrine and paracrine inflammatory loop that underpins systemic cytokine escalation. Thus, this IL-1-IL-6 axis represents a central driver of endothelial dysfunction and systemic toxicity, highlighting CRS as a coordinated failure of adaptive–innate immune crosstalk rather than isolated T cell hyperactivation (14, 19).

Clinically, this cytokine amplification leads to widespread endothelial activation characterised by increased adhesion molecule expression, VE-cadherin disruption, and von Willebrand factor release. These vascular changes drive capillary leakage, hypotension, and coagulopathy, forming the pathological basis of severe CRS (19, 20). Systemic inflammation further leads to multi-organ dysfunction, including myocardial depression mediated by TNF and IL-6, renal hypoperfusion with microthrombus formation, hepatic Kupffer cell activation, and pulmonary endothelial injury, which in severe cases can progress to death (14, 19).

Additionally, metabolic reprogramming sustains this inflammatory state. Activated macrophages undergo hypoxia-inducible factor 1-α (HIF-1α) and mechanistic target of rapamycin (mTOR)-driven glycolytic reprogramming, which reinforces IL-1β production and perpetuates cytokine amplification (21, 22). Concurrently, CAR-T-cells themselves adapt metabolically to support effector function, although inflammatory microenvironments can disrupt this balance, contributing to both toxicity and impaired therapeutic durability (23).

Despite the clinical use of IL-6 receptor blockade (e.g., tocilizumab), CRS management remains largely reactive and incompletely effective in severe cases (14). This limitation reflects persistent upstream inflammatory signalling and redundancy within the cytokine network, which cannot be fully controlled by single-pathway inhibition (24). Moreover, endogenous anti-inflammatory systems, including IL-10 signalling, SOCS proteins, and immune checkpoints, are often overwhelmed during CAR-T activation, resulting in a breakdown of immune homeostasis (25).

Within the IL-1 superfamily, IL-37 and IL-38 have emerged as important anti-inflammatory mediators. Although they have been implicated in diverse inflammatory conditions, including non-small cell lung cancer (26), rheumatoid arthritis (27), and acute pancreatitis (28), their roles in CAR-T-associated CRS remain largely unexplored. A systematic evaluation of current evidence is therefore warranted to define their mechanistic relevance, assess their potential as biomarkers of CRS severity, and explore therapeutic implications. This mini-review integrates CAR-T-driven inflammatory networks with emerging IL-37/IL-38 biology to identify potential endogenous regulatory axes that may inform more precise strategies for CRS control.

## Endogenous immune restraint: IL-37 and IL-38 in CRS regulation

### IL-37: a systemic regulator of pathological inflammation

IL-37 is a broad-spectrum anti-inflammatory cytokine that functions as a key endogenous suppressor of excessive innate and adaptive immune activation. In inflammatory stress states, including CAR-T-associated CRS, it operates at the level of systemic cytokine amplification, acting predominantly on activated monocytes, macrophages, and endothelial cells that drive disease severity.

Mechanistically, IL-37 exerts its effects through coordinated pathways, including inhibition of NF-κB and MAPK signalling, formation of the IL-18Rα–SIGIRR [single immunoglobulin interleukin-1 receptor-related receptor] (IL-1R8) inhibitory receptor complex (29), suppression of macrophage-derived cytokine transcription, and metabolic reprogramming that shifts activated immune cells towards oxidative phosphorylation (30). In the context of CAR-T therapy, IL-37 is likely to attenuate systemic cytokine amplification, particularly IL-6 and TNF production, thereby limiting CRS severity.

IL-37 suppresses CRS-associated inflammation through multiple convergent mechanisms. It inhibits NF-κB and MAPK signalling, thereby reducing the transcription of key pro-inflammatory mediators, including IL-6, TNF, and IL-1β. In addition, IL-37 signals *via* the IL-18Rα-IL-1R8 (SIGIRR) receptor complex, leading to broad suppression of Toll-like receptor (TLR)-driven activation (29). In parallel, it constrains metabolic reprogramming in activated macrophages by limiting glycolysis-dependent cytokine production and attenuates endothelial activation, thereby preserving vascular barrier integrity (29, 31, 32). In the context of CAR-T-treated multiple myeloma, IL-37 can be conceptualised as a system-level dampener of the myeloid amplification loop, acting upstream of the IL-6-dominant cytokine surge that defines severe CRS (18). Given its potent immunosuppressive properties, excessive IL-37 activity may theoretically impair CAR-T-cell effector function by attenuating the inflammatory and cytokine signalling pathways required for sustained anti-tumour immunity and T-cell activation (30), thereby highlighting the importance of maintaining a tightly regulated immunological balance.

### IL-37α as a dominant isoform in lethal cytokine storm control

Experimental evidence from animal models of CRS has demonstrated that among IL-37 isoforms, IL-37α exhibits particularly potent anti-inflammatory activity and serves as a critical determinant of survival in models of lethal cytokine storm. Functional studies demonstrate that silencing IL-37α, or global IL-37 knockdown, increases LPS/IL-1β-induced IL-1α, IL-1β, and IL-6 secretion by approximately 40%, underscoring its essential role in restraining hyperinflammatory responses. Conversely, overexpression of IL-37α in murine macrophages significantly suppresses cytokine production induced by diverse innate immune stimuli, including TLR4, TLR1/2, TLR3, TLR5, TLR8, and IL-1β signalling pathways (33).

*In vivo*, IL-37α provides superior protection against endotoxin-induced lethality compared with IL-37β. Following LPS challenge, wild-type mice exhibit rapid mortality, whereas IL-37β transgenic mice show partial survival benefit. In contrast, IL-37α-transgenic mice demonstrate complete survival at early time points (up to 20 hours), despite mild systemic illness. Consistent with its potent immunomodulatory properties, recombinant IL-37α significantly improved survival (90%) relative to IL-37β (50%) and untreated controls (0%). The observed survival correlated inversely with proinflammatory markers (IL-1α, IL-1β, TNF, and IFNγ), further validating the therapeutic potential of IL-37α in mitigating CRS (33).

Mechanistically, IL-37α exerts protective effects through both extracellular and intracellular pathways. Extracellularly, it signals via IL-18Rα and IL-1R8 (SIGIRR), forming an inhibitory receptor complex that suppresses TLR- and IL-1 receptor-mediated NF-κB and MAPK activation (29). Notably, IL-37α also possesses IL-1R8-independent nuclear activity, distinguishing it from other IL-37 isoforms.

Intracellularly, IL-37α translocates to the nucleus *via* its nuclear localisation sequence and importin-dependent transport, where it functions as a transcriptional regulator. By interacting with Smad3 and promoting PPARγ-dependent transcriptional reprogramming (33), it broadly suppresses pro-inflammatory genes, including IL-6, TNF, and IL-1β. This dual signalling mechanism enables IL-37α to inhibit both inflammatory initiation and cytokine amplification.

IL-37α primarily targets myeloid cells, particularly monocytes and macrophages, key drivers of cytokine storm syndromes (30). By suppressing NLRP3 inflammasome activation and IL-1β maturation, it interrupts early inflammatory amplification that fuels IL-6-dominant cytokine cascades. IL-37α also attenuates endothelial activation, reducing adhesion molecule expression and vascular permeability, thereby preserving vascular integrity and limiting capillary leakage. Supporting its clinical relevance, *ex vivo* studies of paediatric sepsis samples have shown exaggerated inflammatory responses in LPS-stimulated peripheral blood mononuclear cells under conditions of insufficient IL-37 activity (33).

### IL-38 in CAR-T–associated CRS: a phase-aligned regulatory hypothesis

IL-38 is a more recently identified anti-inflammatory member of the IL-1 cytokine family that suppresses both innate and adaptive immune responses (34, 35). Structurally, IL-38 shares substantial homology with IL-36 receptor antagonist (IL-36Ra) and IL-1 receptor antagonist (IL-1Ra), enabling it to modulate signalling through members of the IL-1 receptor family. Current evidence suggests that IL-38 primarily exerts its immunoregulatory effects by binding to IL-36R and, under certain conditions, IL-1RAPL1 and IL-1R1, thereby attenuating downstream NF-κB, MAPK, and AP-1 signalling pathways (36). Through these mechanisms, IL-38 suppresses the production of pro-inflammatory cytokines and chemokines, including IL-6, TNF, IL-17, IL-22, CXCL1, and CXCL2, while promoting a more controlled immune environment (37).

Immunologically, IL-38 acts at the interface of innate and adaptive immunity. In innate immune cells, particularly macrophages, monocytes, dendritic cells, and neutrophils, IL-38 limits inflammatory activation, inhibits NLRP3 inflammasome signalling, and reduces the release of cytokines that drive systemic inflammation (38). In the adaptive immune compartment, IL-38 suppresses the differentiation and effector functions of Th1 and Th17 cells (39) while favouring the expansion and stability of regulatory T cells (Tregs). This shift promotes immune tolerance and restrains excessive tissue-damaging inflammation. Emerging evidence also indicates that IL-38 influences macrophage polarisation by suppressing pro-inflammatory M1 phenotypes and facilitating the development of anti-inflammatory M2 macrophages (40), further contributing to the resolution of inflammation.

Collectively, these properties position IL-38 as an important endogenous regulator of immune homeostasis that functions to limit excessive inflammatory responses while preserving protective host immunity. Dysregulation of IL-38 expression or signalling has been implicated in a broad spectrum of inflammatory, autoimmune, infectious, and cardiovascular diseases, highlighting its potential as both a biomarker and therapeutic target.

In patients with multiple myeloma, circulating IL-38 levels have recently been reported to be significantly reduced compared with those in healthy controls (41), with lower IL-38 expression inversely correlating with disease severity. In contrast, pro-inflammatory cytokines, including IL-1, IL-2, IL-8, and TNF, are markedly elevated in multiple myeloma, suggesting that IL-38 may exert a protective anti-inflammatory role during myeloma progression. Notably, age is a well-recognised prognostic factor in multiple myeloma, with older patients more frequently presenting with higher ISS stage and significantly reduced overall survival (42). Given that age is associated with both disease severity and immune dysregulation in multiple myeloma, it may act as a potential confounding or modifying factor in the observed association between IL-38 levels and disease stage (43).

Despite the absence of direct clinical or preclinical studies evaluating IL-38 in CAR-T therapy for multiple myeloma, its established role as an IL-1 family immunomodulator provides a compelling mechanistic basis for potential involvement in CRS. During the initiation phase of CRS, CAR-T-cell activation and tumour cell lysis lead to the release of damage-associated molecular patterns (DAMPs), which rapidly prime monocytes and macrophages and trigger early inflammatory signalling, including IL-1β production (44). In this setting, IL-38 may function as an endogenous counter-regulatory cytokine within the IL-1 family, attenuating monocyte–macrophage activation and suppressing downstream cytokine amplification through inhibition of innate immune signalling pathways and IL-1/IL-6 axis propagation.

As CRS progresses into the amplification phase, a self-reinforcing inflammatory cascade dominated by IL-1β–IL-6 signalling and macrophage activation drives systemic toxicity. In this phase, IL-38 may dampen the feed-forward inflammatory loop by suppressing pro-inflammatory cytokine production and dampening innate immune cell activation, analogous to pharmacological IL-1 blockade strategies such as anakinra (13).

However, during the late or resolution phase, the immunosuppressive properties of IL-38 introduce a potential trade-off: while contributing to restoration of immune homeostasis, excessive IL-38 activity may impair CAR-T-cell persistence and anti-tumour efficacy (45). This dualistic role is supported by emerging evidence indicating that IL-38 can suppress T-cell-mediated anti-tumour responses, including γδ T-cell activity, suggesting that its temporal dynamics are likely critical in determining overall therapeutic outcomes (44).

Mechanistically, IL-38 regulates inflammation through multiple complementary mechanisms. It acts as an antagonist of the IL-36 receptor axis, thereby limiting downstream inflammatory signalling, while also modulating macrophage polarisation to restrain highly pro-inflammatory phenotypes (35). In addition, IL-38 suppresses dendritic cell activation, reducing antigen-presenting capacity and subsequent immune amplification (46). Unlike the broad systemic influence of IL-37, IL-38 functions as a localised rheostat, fine-tuning inflammatory signalling within specialised niches such as the bone marrow to provide a targeted check against CRS-related hyperactivation.

Although CRS and immune effector cell-associated neurotoxicity syndrome (ICANS) are distinct toxicities following CAR-T-cell therapy, they share overlapping inflammatory mechanisms and frequently co-occur. Current evidence indicates that endothelial activation and dysfunction are central to ICANS pathogenesis (47). Excessive production of inflammatory cytokines, particularly IL-1, IL-6, TNF, and IFNγ, drives endothelial injury, blood–brain barrier (BBB) disruption, and increased vascular permeability, enabling entry of circulating cytokines and immune cells into the central nervous system. IL-1 signalling appears to play a key upstream role, as IL-1 blockade attenuates neurotoxicity and BBB disruption in preclinical models (48). Subsequent microglial activation further amplifies neuroinflammation, leading to encephalopathy, aphasia, seizures, and cerebral oedema (49). Importantly, these processes occur within a broader systemic inflammatory milieu driven by CRS, which provides upstream cytokine and endothelial activation signals contributing to ICANS development.

Given their anti-inflammatory properties, IL-37 and IL-38 may therefore be relevant to both CRS and ICANS. Through suppression of IL-1 family signalling, inhibition of NF-κB- and MAPK-dependent cytokine production, attenuation of endothelial activation, and promotion of regulatory immune responses, both cytokines could theoretically limit BBB dysfunction and neuroinflammatory amplification. However, direct evidence for IL-37 (31) and IL-38 (34) in CAR-T-associated neurotoxicity remains limited, warranting further investigation.

The precise mechanisms underlying the initiation of CRS remain incompletely understood. Emerging evidence suggests that, under physiological conditions, IL-37 and IL-38 contribute to immune homeostasis by restraining innate immune activation and limiting cytokine amplification. CRS develops when these regulatory mechanisms become overwhelmed, leading to uncontrolled innate immune signalling (50). A key initiating event is excessive activation of pattern-recognition receptors (PRRs), particularly Toll-like receptors (TLRs) and the NLRP3 inflammasome, triggered by high pathogen burden, microbial virulence factors, or DAMPs (51). These stimuli trigger sustained activation of NF-κB and MAPK signalling pathways, resulting in the rapid and excessive release of pro-inflammatory cytokines, including IL-1β, IL-6, and TNF.

CRS arises when inflammatory drive exceeds endogenous control. Overwhelming PRR signalling saturates IL-37/IL-38–mediated checkpoints, including IL-1R8- and IL-36R-dependent pathways. In parallel, reduced expression or signalling responsiveness in monocytes and T cells weakens feedback inhibition. Age-, comorbidity-, and sepsis-associated immune reprogramming further promotes a myeloid-dominant inflammatory state with impaired regulatory T-cell activity. Once established, a feed-forward loop driven by IL-6–mediated systemic inflammation, endothelial activation, and vascular leakage sustain immune recruitment and cytokine release (52). At this stage, IL-37 and IL-38 are insufficient to restrain escalation, resulting in CRS characterised by hypercytokinaemia, multi-organ dysfunction, and, in severe cases, shock.

### Integrated IL-37/IL-38 regulatory axis in CRS evolution

The temporal and spatial dynamics of CAR-T-associated inflammation suggest that IL-37 and IL-38 operate within a coordinated but non-redundant immunoregulatory axis. In the early phase of immune activation, IL-38 may limit excessive priming of myeloid and dendritic cells within tumour and bone marrow niches, thereby attenuating initiation of the cytokine cascade. During peak CRS, IL-37 is more likely to function systemically by suppressing circulating inflammatory amplification, particularly IL-6- and TNF-driven endothelial injury. In the resolution phase, IL-37 and IL-38 may act in concert to restore immune homeostasis while preserving residual anti-tumour activity.

From a translational perspective, the IL-37/IL-38 axis offers multiple opportunities for therapeutic exploitation. Early biomarker stratification based on circulating IL-37 and IL-38 levels may enable identification of patients with multiple myeloma at increased risk of severe CRS (41). Therapeutically, recombinant or engineered IL-37/IL-38 proteins (31, 53) could be explored to attenuate excessive inflammatory escalation, while next-generation CAR-T constructs incorporating inducible IL-37 or IL-38 regulatory circuits may enable autonomous control of activation-induced toxicity.

Recent advances in cellular engineering provide additional foundation for this approach. Inducible gene circuits and synthetic signalling systems such as TRUCK platforms (54) and synNotch-based logic-gated receptors (55) allow for antigen-dependent expression of effector genes. These programmable CAR-T architectures provide a conceptual foundation for engineering self-regulating immune responses (56). Collectively, these inducible platforms could incorporate IL-37 or IL-38 circuits to achieve self-limiting immune control, balancing potent anti-myeloma efficacy with controlled inflammatory toxicity in patients with multiple myeloma (57).

We acknowledge that clinical attempts to broadly modulate multiple cytokine networks in CRS have historically been challenging. Our mini-review does not propose immediate therapeutic intervention but instead presents a conceptual framework and hypothesis highlighting IL-37 and IL-38 as potential endogenous regulators of hyperinflammation. Furthermore, we emphasise that targeted strategies to restore or harness these pathways remain speculative and require further mechanistic and translational investigation.

## Conclusion

CAR-T therapy has transformed the treatment landscape of multiple myeloma but remains limited by CRS, a severe inflammatory toxicity driven by a self-propagating cytokine network. IL-37 and IL-38 represent complementary endogenous immunoregulatory cytokines that appear to operate at distinct levels of this inflammatory cascade. IL-38 primarily limits early tissue-resident immune activation and innate immune priming, whereas IL-37 suppresses systemic cytokine amplification, endothelial dysfunction, and metabolic inflammation during later stages of CRS. Reinforcement of this regulatory system through biomarker-guided stratification, recombinant cytokine-based approaches, or engineered CAR-T platforms may provide a rational strategy for reducing CRS severity while preserving anti-tumour efficacy in multiple myeloma.

**Figure 1:**
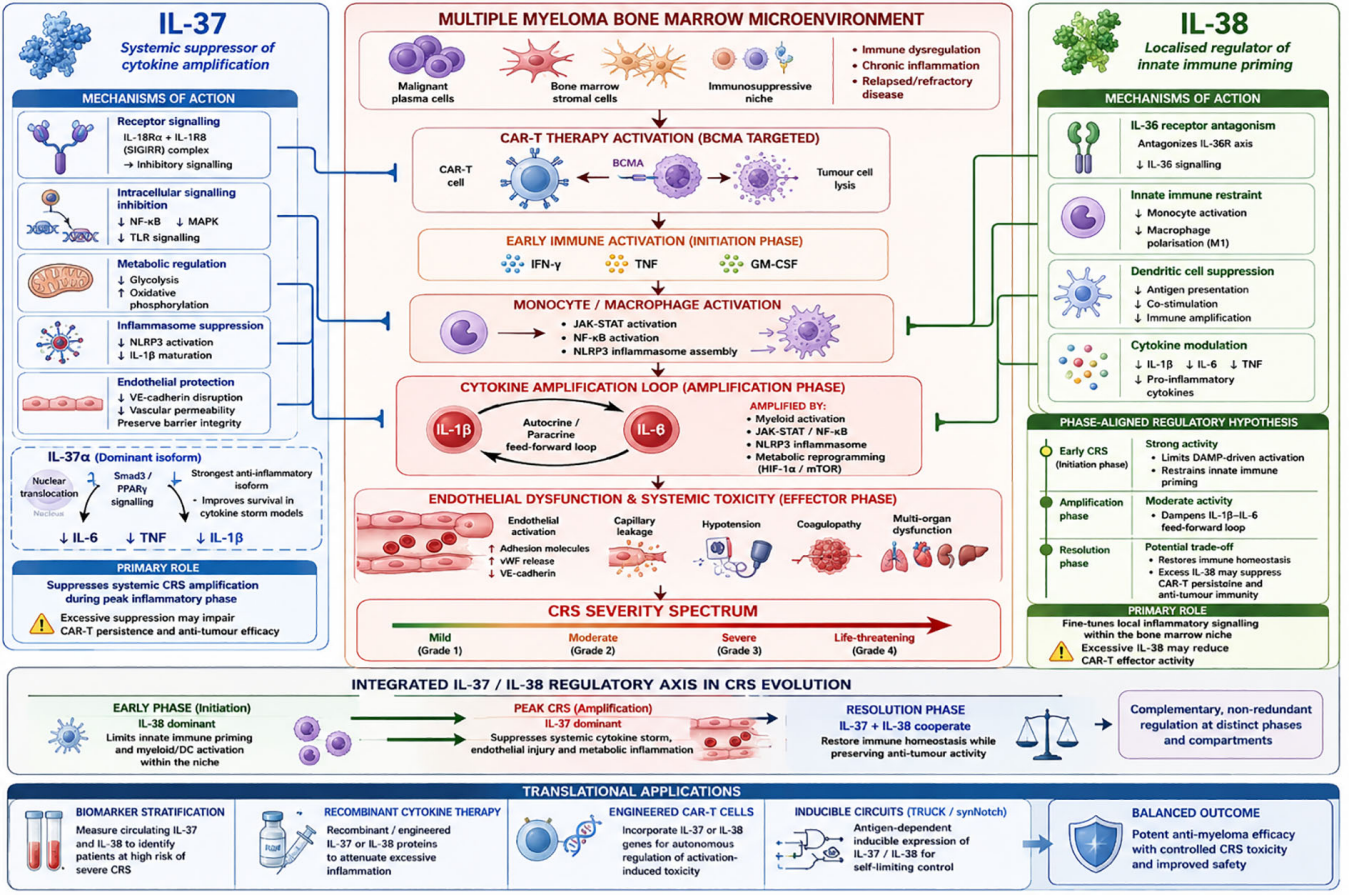
Complementary roles of IL-37 and IL-38 in CAR-T-associated CRS in multiple myeloma.

To summarise the current review, a schematic diagram is presented in **Figure 1** to illustrate the proposed mechanisms and regulatory roles discussed throughout the manuscript. Additionally, a concise summary table comparing IL-37 and IL-38 immunobiology in CRS and ICANS has been included (**Table 1**).

**Table 1:** Comparative immunobiology and translational relevance of IL-37 and IL-38 in CRS and ICANS.

Category	IL-37	IL-38
Inflammatory phase involvement	Early and amplification phases; limits cytokine escalation	Initiation and propagation phases; supports inflammatory resolution
Cellular targets	Monocytes, macrophages, dendritic cells, epithelial cells; indirect T-cell effects	Monocytes, macrophages, dendritic cells; regulates Th1/Th17 and Tregs
Signalling pathways	IL-18Rα/IL-1R8 axis; Smad3/PPARγ nuclear signalling; inhibits NF-κB/MAPK	IL-36R antagonism; suppresses NF-κB/MAPK/AP-1 pathways
Biological functions	Broad anti-inflammatory effects; reduces IL-1β, IL-6, TNF; limits inflammasome activity; protects endothelium	Modulates cytokines/chemokines (IL-6, TNF, IL-17, CXCL1/2); regulates macrophage activation
Immune polarisation	Dampens myeloid inflammation; promotes regulatory tone	Shifts from Th17/M1 toward M2 regulatory/resolving phenotypes
Relevance to CRS	Limits PRR-driven cytokine amplification, IL-6 signalling, and endothelial activation	Reduces Th17/chemokine-driven leukocyte recruitment
Relevance to ICANS	Attenuates BBB disruption via IL-1–mediated endothelial and microglial activation	May reduce CNS chemokine signalling and immune cell infiltration
Translational potential	Biomarker and adjunct to limit CRS severity and ICANS risk	Emerging modulator of systemic inflammatory amplification
Key limitations	Limited clinical translation; context-dependent signalling	Limited mechanistic/clinical validation; receptor complexity

Proposed complementary roles of IL-37 and IL-38 in regulating --associated CRS in multiple myeloma. BCMA-targeted CAR-T-cells trigger inflammatory activation through IFNγ, TNF, GM-CSF, and myeloid-cell-driven IL-1β/IL-6 amplification, resulting in endothelial dysfunction and systemic CRS. IL-38 primarily acts during early CRS by limiting innate immune priming, monocyte/macrophage activation, dendritic cell activation, and IL-36-related signalling within the bone marrow niche. In contrast, IL-37 functions predominantly during peak systemic inflammation by suppressing NF-κB/MAPK signalling, inflammasome activation, endothelial injury, and cytokine amplification. Together, IL-37 and IL-38 form a coordinated endogenous regulatory axis that may provide therapeutic opportunities for biomarker-guided CRS control and engineered self-regulating CAR-T platforms in multiple myeloma.
